# Pilot mental health sensitisation programme for community leaders in Uganda: impact evaluation

**DOI:** 10.1192/bji.2025.10046

**Published:** 2026-02

**Authors:** Linda Shuttleworth, Francesca Pontin

**Affiliations:** 1 Independent Scholar, Chester, UK.; 2 School of Geography, University of Leeds, Leeds, UK

**Keywords:** Low- and middle-income countries, mental health services, impact evaluation, mhGAP, community leader sensitisation

## Abstract

Despite worldwide uptake, there has been little published evaluation of actually delivering the World Health Organization (WHO) Mental Health Gap Action Programme (mhGAP) in typical low- and middle-income countries (LMICs). This paper aims to evaluate the impact of a pilot study in which mhGAP guidelines for mental health sensitisation of community leaders were implemented in 1-day training events across 25 urban and rural health facilities (*n* = 1004 community leaders) in Uganda. A multiple choice mental health questionnaire was used to assess the community leaders’ mental health knowledge before and after completing the training. Training was evaluated across multiple sites and qualitative feedback comments were used to identify key themes on the impact of the training. The sensitisation training was found to be affordable, accessible and effective, and could be replicated in other LMICs and settings with local adaptations.

The World Health Organization (WHO)^1^ estimates that 1 in 8 people globally have a mental disorder; 80% of these individuals reside in low- and middle-income countries (LMICs).^2^ Misunderstanding, stigma and mistreatment of people with mental ill health persists in Uganda as it does across much of the world.^3^ ^,^ ^4^ ^,^ ^5^ ^,^ ^6^ Recognised ways to overcome barriers to mental healthcare in LMICs include gaining political commitment, decentralisation of resources to primary healthcare providers and engaging community members without formal professional training to partake in advocacy and support service delivery.^7^

The Mental Health Gap Action Programme (mhGAP) has been developed by the WHO to scale up services for people with mental, neurological and substance use disorders in LMICs.^8^ Recommendations in the mhGAP Community Toolkit include utilising cost-effective evidence-based interventions and engaging with community providers outside the health sector.^9^ Recognition that education can change community volunteers’ attitudes to people with mental illness, leading to improved treatment and care, has been acknowledged.^10^ However, we have found only one brief description of delivering mental health community awareness events in the context of mhGAP training in the literature,^11^ and no evaluation of impact was reported.

UK registered charity Jamie’s Fund was established in 2013 and over the 10 years of its operation it worked with and through mental health staff and trainers in Uganda to help improve mental healthcare. The charity acted in liaison with the main healthcare providers in Uganda. These are the Mental Health section of the Uganda Government Ministry of Health, and the two faith-based private-not-for-profit health organisations: the Uganda Protestant Medical Bureau (UPMB) and the Uganda Catholic Medical Bureau (UCMB).

Since its establishment, the charity had grown to support 25 health facilities in both urban and rural Uganda to develop mental healthcare for their patients and communities. This had taken the form of funding for equipment (such as computers, motorbikes and solar panels) but more importantly, funding for training. Seven mental health nurses have been supported to undertake psychiatric clinical officer (PCO) training, which equipped them to develop and lead mental health services in their hospitals. Additionally, 1031 non-specialist healthcare staff have acquired fundamental mental health knowledge and skills through the WHO mhGAP training programme, commissioned and funded by Jamie’s Fund.

To address stigma, misunderstanding and consequent suboptimal treatment of those with mental ill health, Jamie’s Fund also embarked on an extensive programme of mental health sensitisation for community leaders who are not healthcare professionals.

This paper aims to evaluate the impact of this Community Leaders Sensitisation programme, using both quantitative data and qualitative comments. Key learning and recommendations are shared to encourage the implementation and evaluation of similar programmes in other LMICs.

## The Community Leaders Sensitisation (CLS) Programme

The WHO developed and published a comprehensive outline programme for community leaders, the mhGAP Community Toolkit, to raise awareness that mental ill health can be recognised as a health condition and treated as such.^9^ It offers an alternative to the commonly held traditional beliefs involving sin, magic, disturbed ancestors and curses as causes.

Three UK Jamie’s Fund clinicians and a senior mental health trainer and researcher in Uganda reviewed the extensive WHO material to produce the content for a 1-day Community Leaders Sensitisation (CLS) event, with a short manual to enable existing mhGAP staff to deliver it. In 2021, JF invited three facilities to trial this 1-day CLS event. Having found it broadly useful and acceptable to the staff and participants, Jamie’s Fund began to invite all its partners to submit plans to run at least one CLS event. The trainers were encouraged to use local languages where appropriate, and to adapt any written material accordingly. As well as general material on understanding mental ill health, the specific topics included were psychosis, depression and epilepsy (in Uganda, because neurology services are scarce, epilepsy is treated within mental health services).

At the time of writing (April 2024), 1004 community leaders from across the country have participated in one of the 25 CLS events (30 days in total, as some facilities ran multiple events), at an average cost of £20 per participant (this covers participants’ and facilitators’ expenses and refreshments, venue hire and materials).

At the request of the CLS trainers, Jamie’s Fund and Ugandan clinicians have since developed substance misuse and suicide prevention as additional topics to add to their repertoire and include as needed.

## Research method

As a condition of receiving funding and support from Jamie’s Fund, the partner health facilities were required to submit their budgeted proposals in advance and to report back after they had held a CLS event. They were asked for basic information, such as numbers of participants and their role in the community, the pre- and post-questionnaire scores, and any comments from trainers and participants. The lead author (L.S.) reviewed the comments as objectively as possible and allocated them to three themes: positives, challenges and suggestions, with subcategories as described below.

The participants were members of their local communities, who had responded to the invitation to attend a day event. They were not professional healthcare workers, nor were they patients of local services. CLS participants represented a wide range of roles within local communities, such as youth leaders, teachers, civic leaders, traditional healers, village health team volunteers, church leaders, community workers, police, boda-boda (motorbike taxi) drivers, social workers, business people, carers, prison officers, traditional birth attendants and representatives of the elderly.

The CLS participants anonymously completed a short questionnaire to test their knowledge and understanding at the start and the end of the day. Their comments and suggestions were also recorded and communicated anonymously. The health facilities hosting the events have also been anonymised. The participants were advised and consented to reporting of the events. We therefore feel confident that formal ethical approval is not required for the publication of this paper.

A pre- and post-event multiple choice questionnaire (MHMCQ) was used to evaluate changes in participants’ mental health knowledge and understanding. It was adapted from a longer questionnaire routinely used to evaluate the effectiveness of mhGAP training for non-specialist health staff^8^ (pp. 152–8). The full MHMCQ is shown in Supplementary Appendix 1, available at https://doi.org/10.1192/bji.2025.10046. Questionnaires were scored by the trainers based on the number of correct answers given. MHMCQ scores were collated and differences in pre-and post-event scores quantified to indicate the effectiveness of the intervention. Seven event participants did not complete a pre- or post-event MHMCQ and a further four event participants failed to complete a post-event MHMCQ. Event participants with missing data were excluded from the quantitative analysis.

All analysis was conducted in Python using the Pingouin (version 0.5.3 for macOS; https://pingouin-stats.org) and scipy.stats (version 1.12.0 for macOS; https://docs.scipy.org) packages. After calculating summary statistics and checking the assumption that the normality of score difference (Shapiro–Wilk test) was met, a paired *t*-test was used to compare pre-scores and post-scores across all facilities. To compare within-facility test score improvements, the Wilcoxon signed-rank test score was calculated, as smaller sample sizes (*n* < 30) meant assumptions of normality could not be made.

## Results

### Pre- and post-training test scores

Twelve sets of pre- and post-event scores were returned, from eight hospitals and health centres. Together these produced 360 sets of participants’ scores, or 35.9% of the total 1004 participants in CLS events at the time of writing.

A paired samples *t*-test was conducted to determine the effect of the sensitisation on the MHMCQ scores. The results indicate a significant difference between test scores before (mean 58.46; s.d. = 18.85) and after sensitisation (mean 72.18; s.d. = 18.8; *t*(360) = −17.07, *P* < 0.01). The 95% confidence interval of the difference between the means ranged from −15.3 to −12.14, indicating a significant difference between the means of the samples. We therefore reject the null hypothesis that there is no difference between the means and conclude that there is a positive effect of sensitisation on the assessment test score.

**Fig. 1:**
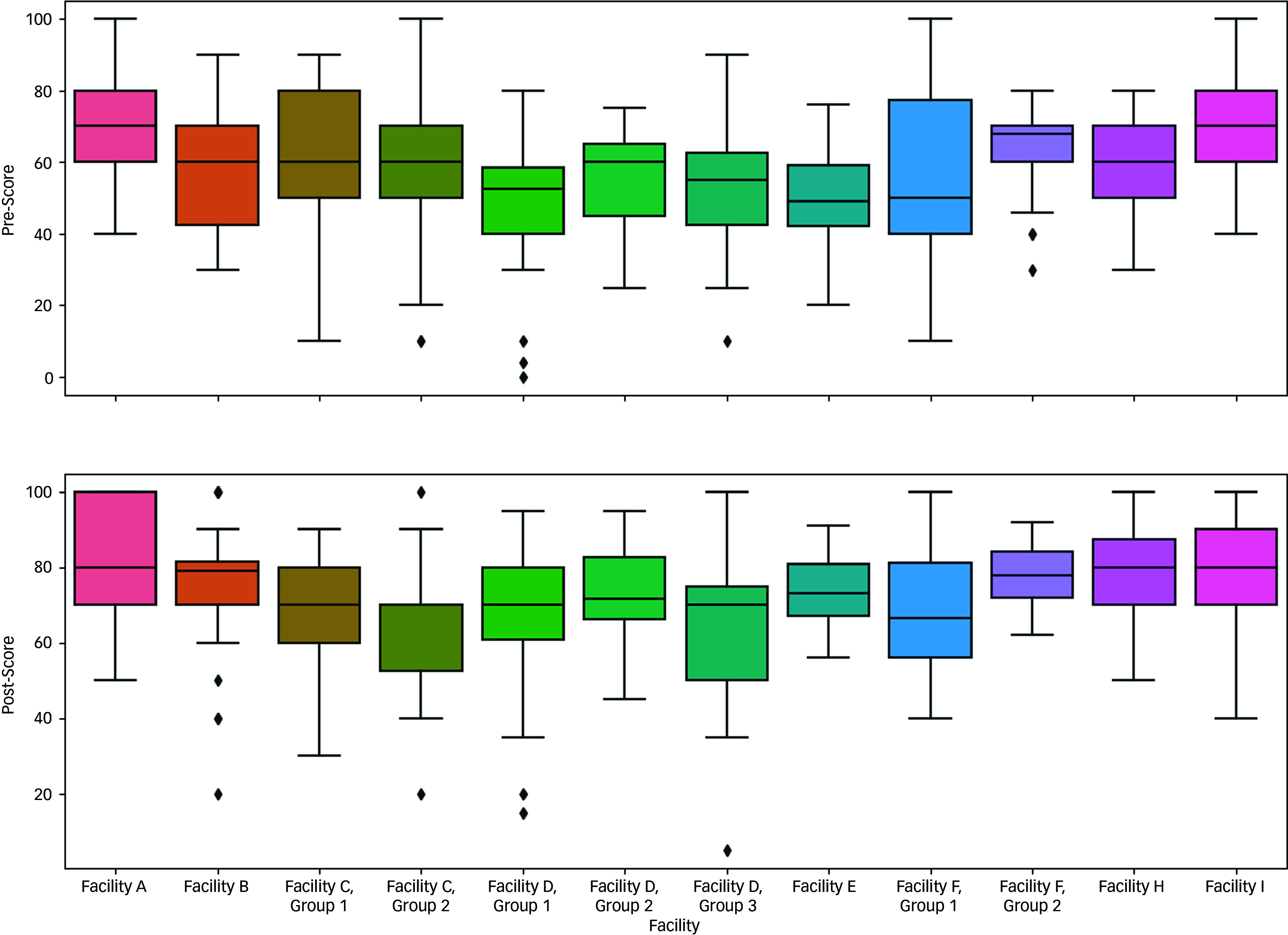
Boxplot of scores pre- and post-delivery of mental health sensitisation, by facility.

Wilcoxon signed-rank tests were conducted to determine the within-facility effect of sensitisation on assessed test scores, all of which were found to be significant (*P* < 0.01), indicating an increase in MHMCQ score across all facilities following sensitisation. Pre- and post-sensitisation scores are illustrated in Fig. 1.

### Qualitative responses

Qualitative responses were extracted from reports of the CLS events (see Supplementary Appendix 2 for examples). Positive comments were made in 21 of the 22 narrative reports received. It was possible to group them into four main themes:

- a changed understanding of mental ill health (11 comments)
- spreading the word (7 comments)
- changed response to people in the community with mental ill health (10 comments)
- increase in referrals (6 comments).

Six of the narrative reports recorded challenges, the themes being:

- challenges in running the CLS events (3 comments)
- challenges to do with mental health services (4 comments).

Eleven of the narrative reports included suggestions or recommendations, the themes being:

- service improvements (3 comments)
- extending the CLS programme (4 comments)
- improving the CLS training and guide (4 comments).

## Discussion

This paper finds evidence for the efficacy of a low-cost 1-day Community Leaders Sensitisation (CLS) event to increase community awareness of mental ill health in Uganda. Mental Health Multiple Choice Questionnaire (MHMCQ) scores showed a significant improvement in mental health knowledge and understanding across all healthcare and community settings where the training was delivered. Moreover, themes identified in follow-up reporting on the successes and challenges in running the training included a positive improvement in the understanding of mental ill health and positive improvement in the response to people in the community with mental ill health.

The community leaders undertaking the training came from a wide variety of roles. The universal improvement in understanding across facilities and sessions where the event was delivered suggests that the materials to support the sensitisation event are adequate to meet the needs of different trainers reaching a variety of audiences. Future research is needed on how best to adapt the training to different community roles.

The success of this training was realised by utilising the existing expertise in local systems and communities.

We identify the following recommendations to adapt similar CLS events to other LMIC settings: use local knowledge to adapt the content and delivery of the WHO mhGAP Community Toolkit, and translate into and deliver in local languages as appropriate, keeping written material easily readable and jargon free.

Qualitative feedback of those running the training also identified further roles of community leaders in better supporting mental health services in Uganda, including: local and national leaders advocating for reliable, available and accessible basic mental healthcare for all citizens; and the need to continue to share positive and accurate messages about mental health across community settings. Future training or workshop sessions are recommended to encourage community leaders to consider how they can use their voice to continue to support and develop community mental health services.

Recommendations from trainers to improve the uptake of future training delivery include: mobilising in more remote locations to effectively optimise attendance; and being creative in using a variety of media to advertise training.

To better support health facilities receiving the training, feedback recommended preparing local providers for possible increased demand on mental health services, and prior scoping of local mental health service challenges to better adapt the training content to the local situation.

### Limitations

There are full sets of MHMCQ scores for only 36% of CLS event participants; they may not be representative. Moreover, a control group was not possible in the context of this pilot study as individuals self-selected to participate in the training to support their communities, and this is the first known mhGAP CLS available in Uganda; thus, the generalisability of these results is limited. Furthermore, the MHMCQ tested understanding of aspects of mental ill health, but has not been formally evaluated as a research tool. However, the literature shows very little reported evaluation even of the mhGAP training for health staff (only 33 studies despite uptake in 90 countries) and the need for standardised evaluation methods.^12^ It is hoped that this pilot study will prompt more systematic and replicable evaluations of mhGAP-based activities.

The qualitative comments recorded in the reports of the various CLS events may have been biased because the reports were returned to representatives of the charity providing funding for the events and other training. They were noted and grouped into themes as objectively as possible, but future evaluations should handle such data using an accepted formal framework for qualitative data.

In conclusion, our experience has shown that health staff trained as mhGAP trainers are able to deliver an effective 1-day Community Leaders Mental Health Sensitisation event at relatively modest cost. The indications are that this increases understanding, challenges stigma and mobilises community action. There is a challenge for government and local services, however, in how to meet the resultant increased demand for mental healthcare.

## Supporting information

Shuttleworth and Pontin supplementary material 1 Shuttleworth and Pontin supplementary material

Shuttleworth and Pontin supplementary material 2 Shuttleworth and Pontin supplementary material

## Supplementary material

The supplementary material is available online at https://doi.org/10.1192/bji.2025.10046
