# Supplementary material for: Pilot mental health sensitisation programme for community leaders in Uganda: impact evaluation
Source: BJPsych Int. 2025 Jul 30;23(1):48–52. doi: 10.1192/bji.2025.10046 (PMC13054145; doi:10.1192/bji.2025.10046)
Supplement: Shuttleworth and Pontin supplementary material 1 — Shuttleworth and Pontin supplementary material [file S2056474025100469sup001.docx]

**Appendix 1**


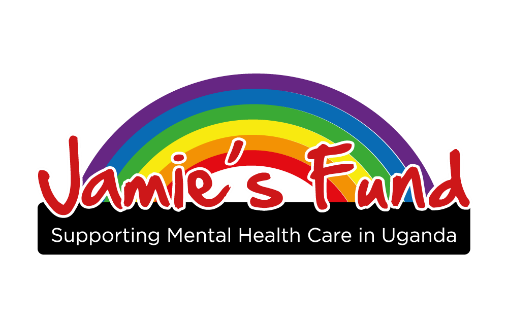
**Pre / post-test for Community Leaders Sensitisation sessions.**

**Name ……………………. Location ……………. Date…………………..**

**1. Which of the following is true of mental illnesses?**

Choose the best answer

1. Mental illness can be treated like any other illness
2. Mental illness can be caught from someone who has it.
3. Mental illness is a disease only of the poor.
4. People with mental illness have been bewitched.
5. Mental illness is caused when the ancestors are angry

**2. Which of the following should regularly happen in your community?**

Choose the best answer

1. Make sure that people with mental illness are kept away from others
2. Ensure that people with mental illness are given medicine to keep them weak or asleep, so that everyone is safe
3. Make sure that all people with mental illness are locked up.
4. Notice the signs of possible mental illness, and link the person to a hospital or health centre.
5. Make sure that only professionally qualified people have conversations with people having mental illness

**3. Which of the following is true of people with a mental illness?**

Choose the best answer

1. All people with mental illness are very dangerous.
2. People with mental illness can often be helped at a hospital
3. People with mental illness are always restless and very noisy.
4. If a parent has mental illness, then the children will also have it.

e) People with mental illness should never get married

**4. Which of the following statements concerning epilepsy is correct?**

Choose the best answer:

1. Epilepsy can be caught from someone who has it
2. Epilepsy is a sign of possession by spirits
3. People with epilepsy cannot hold any job
4. Children with epilepsy can’t go to school and learn like other children
5. Epilepsy is one of the most common disorders of the brain and can be effectively treated

**5. Which of the following statements is true?**

Choose the best answer:

a) The only way to control people with mental illness is to chain them up.

b) You should never allow people with mental illness to control their own money.

c) Giving the right medication for epilepsy is a good way to keep the disease under control

d) Do not expect people with epilepsy to be able to learn anything

e) Do not let children with epilepsy mix with other children, as the others may catch the disease

**6. Which one of the following is good practice when a person is suffering an epileptic attack (seizure)?**

1. Give them something to drink
2. Hold them down to stop the movements
3. Put some object in the mouth to hold it open
4. Make sure they are always kept away from school or work
5. When the seizure is over, make sure that the person has taken medical advice and has medication as needed

**7. Which of the following groups of symptoms best fits with an episode of depression?**

Choose only one answer:

1. Marked behavioural change, disturbed or aggressive behavior, fixed false beliefs
2. Decline in memory, confusion, not knowing where they are or what day it is, not recognising people
3. Inattentive, over-active, aggressive behavior
4. Low energy, sleep problems, loss of interest in usual activities, multiple physical complaints
5. Drinking too much alcohol and spending more money than they have

**8. Which of the following statements concerning severe mental illness is correct?**

Choose the best answer:

1. People with severe mental illness do not need evaluation for physical health conditions
2. People with severe mental illness are best cared for with long-term hospitalization
3. People with severe mental illness are unlikely to be able to work or contribute to society
4. People with severe mental illness are at high risk of stigma and discrimination
5. People with severe mental illness are always at risk of harming others

**9. Which one of the following is a good way to help a person with serious mental illness?**

Choose the best answer:

1. Recommend against work or serious relationships as they may be too stressful
2. Discuss with the carer and family whether long-term hospitalization may be appropriate
3. Teach them things that can help them, especially to get enough sleep, and to avoid stress, drugs and alcohol
4. Discuss with the carer different ways that they might be able to challenge the person’s wrong ideas
5. Expulsion of evil spirits from the person

**10. Which of the following is considered an effective way of talking with someone?**

Choose the best answer:

1. Finishing the conversation as quickly as possible
2. Start by listening carefully to the person
3. Use questions that can be answered with yes or no as it is simpler
4. Not looking at the person much
5. Avoid talking a lot about feelings

April 2021

Supported by Jamie’s Fund <https://jamiesfund.org.uk>

**Correct answers for the Pre / post-test for Community Leaders Sensitisation sessions**

1 a) Mental illness can be treated like any other illness

2 d) Notice the signs of possible mental illness, and link the person to a hospital or health centre.

3 b) People with mental illness can often be helped at a hospital

4 e) Epilepsy is one of the most common disorders of the brain and can be effectively treated

5 c) Giving the right medication for epilepsy is a good way to keep the disease under control

6 e) When the seizure is over, make sure that the person has taken medical advice and has medication as needed

7 d) Low energy, sleep problems, loss of interest in usual activities, multiple physical complaints

8 d) People with severe mental illness are at high risk of stigma and discrimination

9 c) Teach them things that can help them, especially to get enough sleep, and to avoid stress, drugs and alcohol

10b) Start by listening carefully to the person
