# Supplementary material for: Pilot mental health sensitisation programme for community leaders in Uganda: impact evaluation
Source: BJPsych Int. 2025 Jul 30;23(1):48–52. doi: 10.1192/bji.2025.10046 (PMC13054145; doi:10.1192/bji.2025.10046)
Supplement: Shuttleworth and Pontin supplementary material 2 — Shuttleworth and Pontin supplementary material [file S2056474025100469sup002.docx]

**Appendix 2**

**Positive Comments:**

Positive comments were made in 21 of the 22 narrative reports received. It was possible to group them into four main themes:

1. A changed understanding of mental ill health: (11 comments) Examples include ‘…*will reduce stigma’ ‘…leaders were surprised that ‘these people can be helped at hospitals’. ‘A quite number of the leaders were surprised by some features of mental illness especially psychosis and were surprised to know that it can be treated medically without going to the witch doctors as most of them had thought it's actually evil spirits as well as witch craft.’ ‘…we invited 50 participants who all attended the training and at the end of the training appreciated the mental health services, got the knowledge which changed their negative attitude to positive attitude towards mental health.’*
2. Spreading the word: (7 comments) Examples include ‘*Following the training, participants have conducted meetings in their location for awareness creation.’*  ‘*The trained leaders are spreading the gospel in churches and other public places’ ‘It also targeted the right people who in their remarks promised to disseminate the message by all opportunities possible in their respective areas.’*
3. Changed response to people in the community with mental ill health: (10 comments) Examples include ‘*Yesterday we discovered a mental patient in … being locked in the house by the relatives. We assessed the patient and he is now started on antipsychotics. So I have a confidence to identify and manage more mental issues together with the leaders.’ ‘…at the end of the session we made an action plan with the participants, who are expected to respond to violence, exploitation and neglect, and provide PSS (psychosocial support) to families affected with mental disabilities.’ ‘…at the end of the CLS, we had identified 4 community patients… whom their relatives and their leaders had given up on, abandoned and perturbed on what next for their relatives. These are now under our follow-up and control.’*
4. Increase in referrals: (6 comments) Examples include *‘There was an increase in referrals from church leaders and police after the CLS.’ ‘Effective mobilisations were realised by full turn up of all invited community leaders. There was a sudden increase in the turn up of patients seeking mental health (care) after the training.’ ‘… many more people were identified after the sensitisation…’*

**Challenges:**

Six of the narrative reports recorded challenges, the themes being:

1. Challenges in running the CLS events: (3 comments) including lack of English language and literacy in some participants, late and unexpected arrivals, and technical/equipment issues at the venue. Examples include *‘We planned for and invited 60 local community Leaders but ended up with 74 leaders by a yet to be discovered mechanism.’ ‘some arrive late and time is wasted’*
2. Challenges to do with MH services: (4 comments) including inability to afford treatment, and increased referrals and clinic attendances overwhelming the capacity of local services. Examples include *‘Noted … that many of the mentally ill clients are from financially disadvantaged families who do not afford good or improved mental health here … hence end-up in public health facilities…* ***BUT*** *they* ***hardly*** *find treatment there, it is just written to them to* ***BUY*** *from private pharmacies bringing* ***POOR*** *treatment adherence and recovery/improvement issues.’ ‘We realised a large turn up of mentally ill patients on the clinic day after the training…which caused drug stock outs, and many of the clients were not able to pay just consultation.’*

**Suggestions**:

Eleven of the narrative reports included suggestions or recommendations. The themes being:

1. Service improvements: (3 comments) Examples include *‘Start a local Health Insurance Scheme’ ‘consider outreach programmes to support community leaders’ ‘community mental health teams should be established’*
2. Extending the CLS programme: (4 comments) Examples include *‘Consider using local media platforms like radios and TV so that our community which are at long distances can also benefit.’ ‘More trainings for community leaders are needed’ ‘Sensitization among institutes such as schools, markets, universities should be made…’*
3. Improving the CLS training and guide: (4 comments) Examples *include ‘The community mental health guide should include other mental health conditions’ ‘The guide should be translated into local languages.’ ‘Playing the WHO video on the priority MH disorders brought an impact among the leaders as they saw how productive the persons in the video were, and caring for children with behaviour disorders made them realise that quality care is for all.’*
